# Effect of vitamin D deficiency on depressive symptoms in child and adolescent psychiatric patients: results of a randomized controlled trial

**DOI:** 10.1007/s00394-020-02176-6

**Published:** 2020-02-27

**Authors:** Lars Libuda, Nina Timmesfeld, Jochen Antel, Raphael Hirtz, Jens Bauer, Dagmar Führer, Denise Zwanziger, Dana Öztürk, Gina Langenbach, Denise Hahn, Stefanie Ring, Triinu Peters, Anke Hinney, Judith Bühlmeier, Johannes Hebebrand, Corinna Grasemann, Manuel Föcker

**Affiliations:** 1 Department of Child and Adolescent Psychiatry, University Hospital Essen, University of Duisburg-Essen, Virchowstr. 174, 45147 Essen, Germany; 2 Research Institute for the Prevention of Allergies and Respiratory Diseases in Childhood, Department of Pediatrics, Marien-Hospital Wesel, Wesel, Germany; 3 Institute of Medical Biometry and Epidemiology, Philipps-University Marburg, Marburg, Germany; 4 Department of Medical Informatics, Biometrics and Epidemiology, Ruhr University Bochum, Bochum, Germany; 5 Pediatric Endocrinology and Diabetology, Klinik für Kinderheilkunde II, University Hospital Essen, University of Duisburg-Essen, Essen, Germany; 6 Department of Endocrinology, Diabetes and Metabolism, Division of Laboratory Research, University Hospital Essen, University of Duisburg-Essen, Essen, Germany; 7 Children’s Hospital, St. Josef-Hospital, Ruhr University Bochum, Bochum, Germany; 8 Department of Child and Adolescent Psychiatry, University Hospital Münster, Münster, Germany

**Keywords:** Vitamin D, Vitamin D deficiency, Supplementation, Depressive symptoms, Depressive disorder, Mood

## Abstract

**Purpose:**

While observational studies revealed inverse associations between serum vitamin D levels [25(OH)D] and depression, randomized controlled trials (RCT) in children and adolescents are lacking. This RCT examined the effect of an untreated vitamin D deficiency compared to an immediate vitamin D_3_ supplementation on depression scores in children and adolescents during standard day and in-patient psychiatric treatment.

**Methods:**

Patients with vitamin D deficiency [25(OH)D ≤ 30 nmol/l] and at least mild depression [Beck Depression Inventory II (BDI-II) > 13] (*n* = 113) were 1:1 randomized into verum (VG; 2640 IU vitamin D_3_/d) or placebo group (PG) in a double-blind manner. During the intervention period of 28 days, both groups additionally received treatment as usual. BDI-II scores were assessed as primary outcome, DISYPS-II (Diagnostic System for Mental Disorders in Childhood and Adolescence, Self- and Parent Rating) and serum total 25(OH)D were secondary outcomes.

**Results:**

At admission, 49.3% of the screened patients (*n* = 280) had vitamin D deficiency. Although the intervention led to a higher increase of 25(OH)D levels in the VG than in the PG (treatment difference: + 14 ng/ml; 95% CI 4.86–23.77; *p* = 0.003), the change in BDI-II scores did not differ (+ 1.3; 95% CI − 2.22 to 4.81; *p* = 0.466). In contrast, DISYPS parental ratings revealed pronounced improvements of depressive symptoms in the VG (− 0.68; 95% CI − 1.23 to − 0.13; *p* = 0.016).

**Conclusion:**

Whereas this study failed to show a vitamin D supplementation effect on self-rated depression in adolescent in- or daycare patients, parents reported less depressive symptoms in VG at the end of our study. Future trials should consider clinician-rated depressive symptoms as primary outcome.

**Trial registration:**

“German Clinical Trials Register” (https://www.drks.de), registration number: DRKS00009758

## Introduction

The term vitamin D subsumes a group of secosterols, with vitamin D_2_ and vitamin D_3_ being the most relevant forms [1]. Vitamin D has been classified as fat soluble, but in contrast to other nutrients of this group, vitamin D status is not mainly determined by its dietary intake, but by endogenous synthesis from cholesterol precursors in the skin through UVB exposition [2, 3]. Another particularity of vitamin D is the high prevalence of insufficiency in large parts of the general population worldwide [4]. Considering the Institute of Medicine (IoM) cutoffs classifying 25(OH) vitamin D [25(OH)D] levels of 30–50 nmol/l as “potentially at risk for inadequacy” and levels < 30 nmol/l as “at risk for deficiency” [1], vitamin D status seems to be critical in children and adolescents in Germany: according to data from a representative study more than 60% of children and adolescents had 25(OH)D < 50 nmol/l [5].

While the IoM cutoffs primarily refer to the observed effects of vitamin D on bone health and calcium absorption [1], more recent research has raised the question whether vitamin D inadequacy could also have detrimental effects on mental health. A potential role in mental disorders is plausible since the vitamin D receptor as well as vitamin D metabolizing enzymes are expressed in various brain regions [6, 7]. Additionally, a modulation of neuroimmune processes has been postulated as one explanation for a potential role in diverse neurological and mental disorders [8]. Most recently, findings in serotonergic neuronal cell lines of cultured rats indicated that treatment with 1,25(OH)_2_D, i.e., the physiological active metabolite of vitamin D, enhanced serotonin concentrations [9].

In childhood and adolescence, the vast majority of studies examining the association between vitamin D and mental health focused on autism spectrum disorders [10]. First evidence regarding the role of vitamin D in depressive disorders derives from single observational studies [11]. A prospective analysis of the Avon Longitudinal Study for Parents and Children (ALSPAC) cohort revealed that participants with total 25(OH)D < 50 nmol/l at age 10 years had a 20% increased risk of self-reported depressive symptoms at age 14 years, but not at age 11 years [12]. Results from a representative cross-sectional analysis in Germany confirmed these findings, as an inverse association was observed between 25(OH)D levels and emotional problems as indicated by the Strengths and Difficulties Questionnaire (SDQ) [13, 14].

Although a recent uncontrolled intervention study in 940 Iranian adolescent girls showed a significant reduction in depression scores after 9 weeks of vitamin D supplementation [15], randomized controlled trials (RCTs) examining the antidepressant effect of vitamin D supplementation have not been conducted to date in children and adolescents. In adults, systematic reviews and meta-analyses of existing RCTs revealed conflicting results which might be explained by limitations in the study design of the included RCTs. In a meta-analysis, Spedding reported that a beneficial effect was confirmed in six out of seven studies “without biological flaws”, i.e., those studies considering only participants with vitamin D deficiency at baseline and using an adequate dose to achieve vitamin D sufficiency [16]. In contrast, six out of eight RCTs with “biological flaws” did not show an effect [16]. Reviewing common limitations in adult RCTs, we recently concluded that future RCTs in adolescence should consider only patients with baseline vitamin D deficiency, apply a sufficient dose of vitamin D, and focus on a well-defined risk group [10].

Considering these key requirements pertaining to the study design, the aim of the present RCT was to examine the effects of an untreated vitamin D deficiency (placebo) versus an immediately supplemented vitamin D deficiency [with 2640 IU vitamin D_3_ per day (verum)] on depressive symptoms in child and adolescent psychiatric patients with at least mild depression. The main hypothesis was that in unsupplemented patients with vitamin D deficiency [placebo group (PG)], the standard psychiatric treatment would result in significantly lower improvement of BDI-II scores compared to the group with immediate vitamin D_3_ supplementation [verum group (VG)].

## Methods

### Study design and participants

This two-armed, parallel group, double-blind RCT was conducted at the University Hospital Essen between 2016 and 2018 (first patient first assessment: June 2016, last patient last assessment: May 2018). The study was registered at the “German Clinical Trials Register” (https://www.drks.de, registration number: DRKS00009758). Details of the study design are provided elsewhere [17].

Participants were in- or daycare patients aged 11.0–18.9 years with hypovitaminosis D and concurrent (at least) mild depressive symptoms recruited upon admission to the Department of Child and Adolescent Psychiatry, Psychosomatics and Psychotherapy (LVR-Klinikum Essen) at the University Hospital Essen, Germany. A screening assessment (T0) for study eligibility was conducted within the first 2 days after hospital admission. Inclusion criteria were a vitamin D deficiency, i.e., 25(OH)D ≤ 30 nmol/l [equivalent to ≤ 12 ng/ml] and a BDI–II sum score > 13 indicating at least mild depression. Exclusion criteria were a concurrent diagnosis of a severe somatic disease, renal disease, hypocalcemia and/or elevated blood plasma parathyroid hormone (PTH) > 130 ng/ml), and/or mental retardation (IQ < 70). Patients with vitamin D deficiency and established hypocalcemia or PTH level > 130 ng/ml were excluded and referred to pediatric endocrinology for further treatment. Children and adolescents meeting all eligibility criteria were allocated to receive placebo or verum for a period of 28 days at the latest on the 5th day after admission.

### Intervention and randomization

All participants were treated according to best clinical practice and existing treatment guidelines for their respective mental disorders, i.e., treatment as usual (TAU). Participants were 1:1 randomized into one of two study groups. Group 1 (VG) received TAU and oral vitamin D_3_ supplementation at a dose of 2640 IU/day, i.e., 66 mg/day, while group 2 (PG) received placebo during the intervention period besides TAU. The selected dose of vitamin D supplementation is substantially higher than the minimum effective dose of 600–800 IU/day described in the meta-analysis of Spedding [16] and also higher than the minimum dose used in the RCTs included in the major depressive disorder (MDD) meta-analysis of adult patients (i.e., 1500 IU/day) [18, 19]. Considering the mean length of hospitalization of inpatient adolescents at our clinic, as well as previous research on pharmacokinetics of vitamin D which indicates the reach of a plateau concentration after 1 month of supplementation [20], the intervention period was set to 28 days. Participants who were discharged from hospital prior to day 28 were asked to continue supplementation in the scheduled manner, followed up weekly by phone, and scheduled for the end-of-study examination on day 28 after randomization (*n* = 18). A vitamin D supplementation with 1000 IU daily was recommended to all participants with persistent vitamin D deficiency at the end of the study.

Vitamin D or placebo was administered orally as pills (880 IU vitamin D_3_/pill), delivered by the same manufacturer (Dr. B. Scheffler Nachf. GmbH & Co. KG, Bergisch Gladbach, Germany) in identical dispensers, labeled with the randomization number. Participants in both groups were advised to take three pills per day in the morning after breakfast. Participants, parents, therapists, and outcome assessors were blinded regarding study allocation.

Randomization was conducted by the Institute of Medical Biometry and Epidemiology [(IMBE), Philipps-University Marburg, Marburg, Germany] using computer-generated random number lists with random block sizes of lengths 2, 4 and 6. The randomization lists were stratified for two BDI-II strata (total score values 14–23 and ≥ 24), respectively [21], and two 25(OH)D strata (low stratum: 25(OH)D < 12.5 nmol/l, high stratum: 25(OH)D between 12.5 and 30 nmol/l) to guarantee an equal distribution in both study groups.

Prior to the study start, randomization lists (numbers and treatment allocations) generated by the IMBE were sent to the company providing the vitamin D/placebo supplements for an appropriate labeling of the pill dispensers. Upon recruitment of a patient, a fax was sent to the IMBE, which then provided information on the individual randomization number of the participant to the study center where the corresponding pill dispenser was assigned.

### Outcome and covariate assessments

The Beck Depression Inventory (BDI)-II [22] sum score at the end of the study was defined as the primary outcome and 25(OH)D concentrations and DISYPS-II DES (Diagnostic System for Mental Disorders in Children and Adolescents according to ICD-10 and DSM-IV, [23]) depression scores—all at the end of study—as secondary outcomes.

BDI-II was used to assess depressive symptom severity at T0 and after 28 interventional days (T1). The BDI-II is a self-reported questionnaire including 21 items covering DSM-IV diagnostic criteria for MDD. Using a four-point Likert scale (0–3 points) with higher scores indicating a greater degree of depression, the BDI-II asks how much these statements describe the participant’s symptoms in the preceding 2 weeks. Kumar and colleagues stated that the items of BDI-II address all nine DSM-IV criteria for a major depressive episode [22]. These are depressed mood, diminished interest or pleasure in most activities, significant weight change or change in appetite, insomnia or hypersomnia, psychomotor agitation or retardation, fatigue or loss of energy, feelings of worthlessness or excessive or inappropriate guilt, diminished ability to think or concentrate, and suicidality. In an adolescent sample of 105 male and 105 female outpatients between 12 and 18 years, Steer et al. observed a high internal consistency of the BDI-II (Cronbach’s alpha = 0.92) [24]. Total BDI-II scores > 13 indicate at least mild depression [22]. The above-mentioned BDI strata were a priori defined considering mean values observed in a German sample of adolescent psychiatric patients [21].

Depressive symptoms at T0 and T1 were additionally assessed using DISYPS-II DES (self- and parent rating). In general, DISYPS-II is a German diagnostic system including self-rating (SBB) and rating by parent and/or teacher (FBB) to support the diagnosis of psychiatric disorders in children and adolescents according to ICD-10 and DSM-IV [23]. The parent-reported questionnaire DISYPS-II FBB and the self-rated version DISYPS-II SBB, respectively, include 29 items covering the ICD-10/DSM-IV criteria for MDD. Using a four-point Likert scale (0–3 points) with higher scores indicating a greater degree of the respective symptom, the questionnaire probes how much these statements describe the participant’s symptoms. Examples for these items are “seems sad most of the time”, “seems grumpy, irritable and cranky most of the time”, “is not interested or derives no pleasure from nearly anything or anybody”, and “seems tired, without energy and exhausted most of the time”. Analyses of internal consistency of DISYPS-II DES revealed a Cronbach’s alpha of 0.89 for the depression scale of both DISYSPS-II SBB and FBB [23]. Total scores were transferred to stanine scores using age- and sex-specific reference values. A stanine score ≥ 7 indicates at least borderline abnormality. Psychiatric diagnoses at T0 were assessed via the semi-structured interview “Kiddie Schedule for Affective Disorders and Schizophrenia for School Aged Children—Present and Lifetime Version” aged 6–18 years (K-SADS-PL) according to DSM-IV [25].

At T0 and T1, blood samples were obtained from an antecubital vein through a short catheter in Monovettes (Sarstedt, Germany) at early morning after an overnight fast. The blood samples were transferred within an hour after blood sampling to the central laboratory of the University Hospital Essen for analyses of serum 25(OH)D, calcium levels and plasma PTH. Whole blood was centrifuged (3350 × g, 10 min, 4 ˚C) after coagulation and serum/plasma was distributed in small aliquots. The study protocol envisaged the same procedure—if possible—in case of a study dropout for any reason.

Serum total 25(OH)D and plasma PTH concentrations were determined with the Siemens ADVIA Centaur® Immunoassay-system (Siemens Healthineers, Erlangen, Germany). The ADVIA Centaur® Vitamin D Total Assay is a competitive immunoassay and according to the product insert the intra-assay variation was < 11.9%, the inter-assay variation was < 5.3%, the functional sensitivity was 4.2 ng/ml (10.5 nmol/l) and the limit of detection was 3.2 ng/ml (8.0 mmol/l). The ADVIA Centaur® Vitamin D Total Assay is a standardized laboratory measurement of 25(OH)D of the NIST (National Institute of Standards and Technology). The ADVIA Centaur® PTH Test is a two-side sandwich chemiluminescence immunoassay and, according to the product insert, the intra-assay variation was < 6.8%, the inter-assay variation was < 5.2%, the functional sensitivity was 4.6 pg/ml (0.488 pmol/l) and the limit of detection was 3.2 pg/ml (0.339 pmol/l). Serum calcium was analyzed with the ADVIA2400 (Siemens Healthineers, Erlangen, Germany) using the colorimetric *o*-cresolphthalein method with an intra-assay variation < 2.4%, an inter-assay variation < 2.0% and a functional sensitivity of 1.0 mg/dl (0.25 mmol/l). The controls were performed according to the product inserts (quality control advice of the manufacturer). Serum 25(OH)D, plasma PTH and serum calcium analyses are accredited according to DIN EN ISO 15189:2014.

Clinical assessments at admission included assessments of general health and parameters such as current body weight and pubertal status.

### Statistical analysis

Sample size calculation with a two-sided Student's *t* test resulted in a required size of 81 patients per group to detect a difference of 4 points in the BDI-II, assuming an SD of 9 points, to attain an 80% statistical power, at a two-sided α of 0.05. The detectable difference for the BDI-II of four points corresponds to the observed effect in an RCT of vitamin D supplementation in depressed adults [26]. Information on standard deviations of the BDI-II in adolescents was derived from RCTs in adolescents with MDD, irrespective of vitamin D as the treatment item [27, 28]. Since a dropout/loss to follow-up rate of approximately 20% was assumed, 100 patients per group should be recruited into the study.

The recruitment period was a priori scheduled to be completed within 2 years. In practice recruitment turned out to be less successful as planned, resulting in a sample of 113 participants (including 13 participants who were “lost to follow-up”) after 2 years of recruitment. Therefore, it was decided to perform an unplanned interim analysis. Before conducting this analysis, it was scheduled to stop the study for futility if the observed difference in the BDI-II reduction was below two points. In addition, sample size modification would be allowed at the interim analysis, where control of the type I error rate would be ensured through the use of the CRP principle [29]. Recruitment of patients was stopped and end of follow-up was awaited for all recruited patients before interim analysis was performed. At the analysis an estimated difference in the BDI-II reduction below 0 was observed, which led to a futility stopping of the trial and therefore recruitment of patients was terminated. Here, we provide results from this interim analysis.

The main analysis considered subjects with complete data and examined all participants within their randomized group (i.e., modified intention-to-treat (ITT) population). Intervention effects for primary and secondary outcomes were analyzed using a linear model (ANCOVA) with the respective outcome variable at the end of the study as dependent variable, and the individual study group, age, sex, 25(OH)D level at baseline and the value of the outcome variable at baseline as independent (co-)variables. Besides BDI-II sum scores at the end of the study, separate ANCOVA models considered the secondary outcomes serum 25(OH)D concentrations, DISYPS-FBB and DISYPS-SBB depression scores at the end of the study as dependent variables to evaluate whether the dietary intervention resulted in significant differences between study groups.

Sensitivity analysis for the primary outcome was conducted with missing values (*n* = 13) imputed via a linear regression model, which predicted the missing BDI-II values from the variables age, sex, BDI and 25(OH)D level at admission [30]. Furthermore, explorative analyses were performed to examine whether antidepressant effects depend on severity of depressive symptoms at baseline. For this purpose, an interaction term (group × BDI-II at baseline) was included as an additional co-variable in the above-mentioned ANCOVA model for BDI-II. An additional exploratory analysis examined the association between the change in BDI-II between T0 and T1 (outcome) and the concomitant change in 25(OH)D levels (exposure) by linear regression, considering 25(OH)D at baseline, BDI at baseline, and age and sex as independent (co-)variables.

Analysis of the data was conducted after database locking with “R” (www.r-project.org, version 3.5.1). For all analyses, two-sided tests were used and *p* values < 0.05 were considered significant.

## Results

### Sample characteristics

**Fig. 1:**
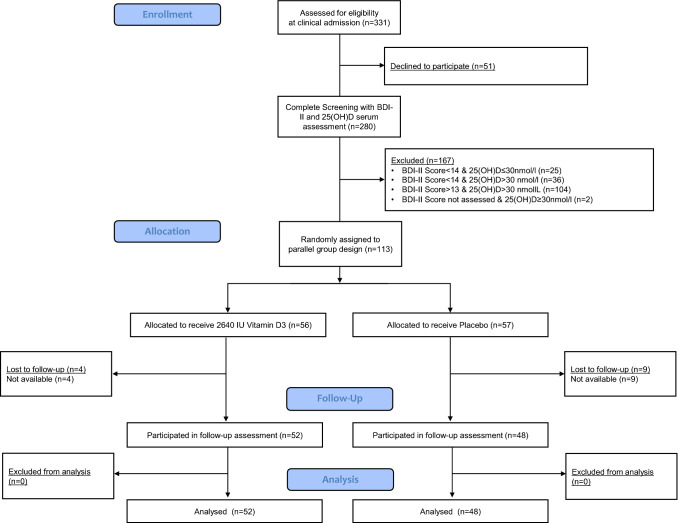
Flowchart of study enrollment, randomization and loss to follow-up during the intervention period

While a vitamin D deficiency was diagnosed in 138 of 280 screened patients (49.3%), 113 (40.4%) participants fulfilled all inclusion criteria and were randomized to VG or PG (Fig. 1). During the intervention period, 13 participants were “lost to follow-up” resulting in a sample of 100 participants with complete data sets for the modified intent-to-treat analysis. Baseline characteristics of randomized patients revealed a similar distribution in both study groups (Table 1): In both, 75% of the participants were females, more than 95% were in the higher vitamin D stratum and nearly 70% were in the higher BDI-II stratum, indicating a moderate to severe depressive episode at T0. Over 90% of the randomized subjects had a KIDDIE-SADS diagnosis of MDD.

**Table 1:** Baseline characteristics of randomized participants

	Placebo group (*n* = 57)		Verum group (*n* = 56)	
	*n*	Mean (± SD) or %	*n*	Mean (± SD) or %
Age [years]	57	15.8 ± 1.65	56	16.1 ± 1.41
Females [*N*, %]	43	75.4%	42	75.0%
Body height [cm]	57	167 ± 8.50	56	167 ± 9.54
Body weight [kg]	57	63.7 ± 18.2	56	67.1 ± 18.9
BMI [kg/m^2^]	57	22.8 ± 5.5	56	23.8 ± 5.9
MDD diagnosis [%]^a^	54	96.4%	51	94.4%
Serum 25 (OH) vitamin D				
Absolute concentration [ng/ml]^b^	57	8.56 ± 2.05	56	8.66 ± 1.97
Stratum 1 (0–5 ng/ml) [*N*; %]	2	3.51%	1	1.79%
Stratum 2 (5–12 ng/ml)	55	96.5%	55	98.2%
BDI-II				
Total scores	57	29.2 ± 9.38	56	31.1 ± 11.3
Stratum 1 (14–23)	18	31.6%	17	30.4%
Stratum 2 (24–64)	39	68.4%	39	69.6%
DISYPS-stanine depression				
Self-report	56	8.54 ± 1.13	54	8.46 ± 1.02
Parent report	54	8.37 ± 1.23	55	8.65 ± 0.82

*BDI-II* Beck Depression Inventory-II, *DISYPS* Diagnostic System for Mental Disorders in Childhood and Adolescence, *MDD* major depressive disorder

^a^Diagnostic and Statistical Manual of Mental Disorders, fourth edition (DSM-IV) major depression disorder diagnosis based on KIDDIE-SADS Present and Lifetime Version (KIDDIE-SADS not available: VG: *n* = 2, PG: *n* = 1)

^b^1 ng/ml 25(OH) vitamin D equates to 2.5 nmol/l

### Effects of vitamin D supplementation on primary and secondary outcomes

Mean 25(OH)D concentrations substantially increased during the intervention in VG, resulting in a higher mean serum 25(OH)D at T1 in VG than in PG (estimated difference verum-placebo: + 14 ng/ml; 95% CI 4.9 to 23.8; *p* = 0.003, Table 2). However, BDI-II scores improved similarly in both groups (PG: − 5.2 ± 8.8; VG: − 4.3 ± 8.5, Table 2) and did not differ between groups at T1 (estimated difference: + 1.3; 95% CI − 2.2 to 4.8; *p* = 0.466, Table 2). Sensitivity analysis yielded similar results with the imputed data set (estimated difference = 0.49; 95% CI − 2.52 to 3.58; *p* = 0.75). The exploratory analysis revealed no evidence for effect modification according to BDI-II scores at T0 [interaction term (group × BDI): β = − 0.003; 95% CI − 0.36 to 0.35; *p* = 0.985]. Additionally, no linear association was observed between the change in 25(OH)D between T0 and T1 and the concomitant BDI change (*β* = 0.049; 95% CI − 0.16 to 0.2; *p* = 0.6415).

**Table 2:** Changes in primary and secondary outcomes during study participation and treatment effect of 4 weeks vitamin D supplementation (verum) compared to placebo

	Placebo group	Verum group	Difference verum–placebo^a^	95% CI^a^	*t* value	*p* value^a^
	*N* = 48	*N* = 52				
BDI-II						
T1	24.4 ± 12.6	27.1 ± 14.0	1.29	− 2.22 to 4.81	0.731	0.466
T1–T0	− 5.21 ± 8.78	− 4.33 ± 8.51				
25(OH) Vitamin D [ng/ml]^b^						
T1	10.4 ± 4.31	23.9 ± 6.11	14.3	4.86 to 23.77	3.005	0.003
T1–T0	1.84 ± 4.24	15.1 ± 6.16				
DISYPS-stanine depression						
Parent report						
T1	8.23 ± 0.95	7.65 ± 1.74	− 0.68	− 1.23 to − 0.13	− 2.458	0.016
T1–T0	− 0.17 ± 1.11	− 0.98 ± 1.69				
self-report						
T1	8.21 ± 1.18	7.96 ± 1.36	− 0.20	− 0.70 to 0.31	− 0.781	0.437
T1–T0	− 0.33 ± 1.55	− 0.48 ± 1.43				

*BDI-II* Beck Depression Inventory-II, *DISYPS* Diagnostic System for Mental Disorders in Childhood and Adolescence, *DISYPS* parent report at T0 not available for *n* = 1 in PG

^a^Linear model (ANCOVA) adjusted for age, sex, baseline vitamin D and baseline value

^b^1 ng/ml 25(OH) vitamin D equates to 2.5 nmol/l

In accordance with the results for the self-rated BDI-II, the self-reported DISYPS-SBB also revealed no group differences in depressive symptoms at T1. In contrast, parent-reported DISYPS-FBB stanine scores at T1 were lower in VG compared to PG (estimated difference: − 0.68; 95% CI − 1.23 to − 0.13; *p* = 0.016; Table 2).

## Discussion

This study is the first RCT on the effect of an untreated vitamin D deficiency on depressive symptoms in children and adolescents and considered several intensively discussed limitations of previous RCTs: As postulated, our study focused on participants who were both vitamin D deficient and at least mildly depressed at baseline [31]. The main finding of this RCT was that an immediate vitamin D_3_ supplementation in depressed child and adolescent psychiatric patients with vitamin D deficiency did not result in a significant decrease of self-reported depressive symptoms, but in a significant decrease of parent-reported depressive symptoms after 4 weeks of in- or day-patient treatment compared to placebo.

These findings are in line with the overall equivocal results from previous studies: observational studies in adolescents indicate an inverse association between 25(OH) D levels and emotional problems [13, 14] or depressive symptoms [12]. Pooled data from observational studies in adults confirmed this association: While nine cross-sectional studies revealed a moderately higher risk of depression for the lowest compared to the highest vitamin D category (OR 1.31; 1.0–1.71 95% CI), three cohort studies even showed more definite associations between vitamin D categories at baseline and the risk of developing depression over time (lowest vs. highest vitamin D category: pooled HR 2.21; 1.40–3.49 95% CI) [32]. These cohort studies did not reveal a definite cutoff for 25(OH)D concentrations which might be required to experience beneficial effects on depression [32]. In contrast, Mendelian randomization studies did not indicate a causal relationship between 25(OH)D concentrations and depression [33, 34].

Meta-analyses of RCTs in adults also revealed no general impact of vitamin D supplementation on depression scores or depressive symptoms [31, 35, 36]. Spedding argued that these null effects might result from “biological flaws” in the design of included RCTs [16]. In the current study we considered the limitations depicted by Spedding [16], e.g., focusing on patients with diagnosed vitamin D deficiency and depressive symptoms as indicated by BDI-II.

While the present study failed to show a significant effect of vitamin D on the primary outcome parameter (BDI, i.e., self-reported depressive symptoms), parent reports of depressive symptoms revealed beneficial effects of vitamin D supplementation. Whereas we cannot dismiss this effect as a spurious finding, we in retrospect and upon review of pharmacological and/or psychotherapeutic RCTs to treat depression in children, adolescents and adults deem it important to point out that our use of the BDI-II as primary outcome could well represent the main reason for the observed conflicting results. Although the BDI-II is commonly used in depression research and served as primary outcome in three of four studies [26, 37, 38] included in the most recent MDD meta-analysis of vitamin D effects on depression [19], self-reports might be less appropriate as primary outcome measure in RCTs focusing on antidepressant effects. Indeed, using the parent-rated DISYPS-FBB as outcome variable we detected a beneficial effect of vitamin D supplementation of 0.7 stanine scores on parent-reported depressive symptoms. As the negative results of the self-rated DISYPS-SBB were in accordance with those for the BDI-II, these conflicting findings seem to rely on the differing raters.

In general, only moderate correlations have been observed between self- and parental-rated mental health of adolescents; furthermore, differences between informants increase with age in particular for internalizing problems [39]. In adults, the depression severity assessed via BDI also correlates only moderately with clinicial ratings using Hamilton Depression Rating Scale [40]. RCTs focussing on the effects of pharmacological antidepressants and/or psychotherapy have revealed larger effect sizes for both verum and placebo when clinician-based ratings were considered in comparison to self-ratings [41-43]. Accordingly, we recommend that future RCTs should include a clinician rating, ideally as the primary outcome as usual in pharmacotherapy trials, to answer the question whether vitamin D supplementation has antidepressant effects.

Another explanation for our negative primary outcome relies on the intervention period of only 4 weeks. This time frame represents the mean hospitalization duration of adolescents in our clinic and, thus, reflects real-life conditions in child and adolescent psychiatry. Anyhow, this time frame might have been too short to cause substantial modifications in brain metabolism and, thus, antidepressant effects. Indeed, a meta-analysis indicated differences in pooled effect sizes for depression between RCTs with follow-ups less than 8 weeks and those with at least 8 weeks [31]. Also in the most recent meta-analysis showing a significant beneficial effect of vitamin D supplementation on MDD, only RCTs with follow-up between 8 and 52 weeks were included [19]. In comparison, RCTs including pharmacological antidepressants were able to show a treatment response after 2–4 weeks [44]. As there is currently no rationale for defining a minimal duration needed for significant antidepressant effects of vitamin D, our study only allows to draw conclusions regarding missing short-term effects of vitamin D supplementation on self-rated depression, but it is not suited to exclude beneficial longer-term effects. Additionally, it has to be kept in mind that vitamin D supplementation was not used as monotherapy in our study, but in addition to TAU which has a separate effect on depressive symptoms. An unbiased examination of the vitamin D supplementation effect was achieved by using TAU in both study groups. However, our study only allows to draw conclusions regarding vitamin D supplementation as adjunctive therapy, but not as monotherapy for depressive symptoms.

The heterogeneity of depression and depressive symptoms as well as of their specific underlying pathophysiologic mechanism could represent another explanation for the lack of a positive finding in our studies. Furthermore, the antidepressant effects of vitamin D supplementation may be particularly apparent at more severe stages of depression. In line with this hypothesis, Shaffer and colleagues observed statistically significant effects only in a subgroup analysis of two studies in participants with clinically significant depressive symptoms, while five studies without clinically significant depressive participants did not show significant effects [36]. We, therefore, examined whether the intervention effect differed according to BDI scores at baseline, but did not observe a significant interaction. Accordingly, we cannot confirm differing vitamin D effects according to baseline depression severity in our study sample.

An alarming finding was the high prevalence of vitamin D deficiency (and potential consequences especially for calcium metabolism and bone health) in all patients screened for this study. A direct comparison with the prevalence in the general adolescent population in Germany is hampered by the fact that a slightly lower cutoff for vitamin D deficiency was used in the most recent representative study KIGGS in Germany (i.e., 25 nmol/l). Although both studies used chemiluminescence immunoassay technology (DiaSorin in KiGGS), differences in vitamin D analytics also make a direct comparison difficult. With these limitations in mind, the prevalence of vitamin D deficiency in our study was high, since nearly 50% of all screened patients had 25(OH)D levels ≤ 30 nmol/l. In fact, 98 of the 280 screened patients (35.0%) were below the cutoff of 25 nmol/l applied in the representative KiGGS study, which applied to only 20% of the participants from the general population [5]. Likewise, 117 adult psychiatric outpatients in Sweden were examined with considerable lower median 25(OH)D levels than reported in samples from healthy Swedish populations [45]. Further studies are needed to confirm whether patients from psychiatric clinics might be a specifically vulnerable group for vitamin D deficiency to draw conclusions whether analysis of 25(OH)D levels should be integrated in psychiatric clinical routine diagnostic.

In conclusion, results on potential antidepressant effects of vitamin D supplementation remain conflicting. While self-rated depression improved similarly in both the verum and the placebo groups of this study, the observed differences in parent-reported depressive symptoms in favor of vitamin D supplementation warrant attention. Therefore, further RCTs in children and adolescents are needed which should include a blinded clinician rating as the primary instrument to assess depressive symptoms. Furthermore, a study duration of more than 4 weeks would appear helpful to allow detection of potential longer-term antidepressant effects of vitamin D supplementation. Considering the fact that almost 50% of the patients were found to be vitamin D deficient, a routine screening in child and adolescent psychiatric patients needs to be debated further.
