# Correction: Effect of vitamin D deficiency on depressive symptoms in child and adolescent psychiatric patients: results of a randomized controlled trial

**DOI:** 10.1007/s00394-025-03748-0

**Published:** 2025-07-21

**Authors:** Lars Libuda, Nina Timmesfeld, Jochen Antel, Raphael Hirtz, Jens Bauer, Dagmar Führer, Denise Zwanziger, Dana Öztürk, Gina Langenbach, Denise Hahn, Stefanie Ring, Triinu Peters, Anke Hinney, Judith Bühlmeier, Johannes Hebebrand, Corinna Grasemann, Manuel Föcker

**Affiliations:** 1 Department of Child and Adolescent Psychiatry, University Hospital Essen, University of Duisburg-Essen, Virchowstr. 174, 45147 Essen, Germany; 2 Research Institute for the Prevention of Allergies and Respiratory Diseases in Childhood, Department of Pediatrics, Marien-Hospital Wesel, Wesel, Germany; 3 Institute of Medical Biometry and Epidemiology, Philipps-University Marburg, Marburg, Germany; 4 Department of Medical Informatics, Biometrics and Epidemiology, Ruhr University Bochum, Bochum, Germany; 5 Pediatric Endocrinology and Diabetology, Klinik Für Kinderheilkunde II, University Hospital Essen, University of Duisburg-Essen, Essen, Germany; 6 Department of Endocrinology, Diabetes and Metabolism, Division of Laboratory Research, University Hospital Essen, University of Duisburg-Essen, Essen, Germany; 7 Children’s Hospital, St. Josef-Hospital, Ruhr University Bochum, Bochum, Germany; 8 Department of Child and Adolescent Psychiatry, University Hospital Münster, Münster, Germany

**Correction to: European Journal of Nutrition (2020) 59:3415–3424** 10.1007/s00394-020-02176-6

The original version of this article included a typing error in the unit of the intervention dose described in the section "Intervention and randomization" of the third sentence. Third sentence, which previously read as "Group 1 (VG) received TAU and oral vitamin D3 supplementation at a dose of 2640 IU/day, i.e., 66 mg/day, while group 2 (PG) received placebo during the intervention period besides TAU" but should have read as "Group 1 (VG) received TAU and oral vitamin D3 supplementation at a dose of 2640 IU/day, i.e., 66 μg/day, while group 2 (PG) received placebo during the intervention period besides TAU".

